# Swiss Priority Setting on Implementing Medication Adherence Interventions as Part of the European ENABLE COST Action

**DOI:** 10.3389/ijph.2022.1605204

**Published:** 2022-08-12

**Authors:** Carole Bandiera, Janette Ribaut, Alexandra L. Dima, Samuel S. Allemann, Kate Molesworth, Kabeza Kalumiya, Fabian Käser, Melvin Skip Olson, Michel Burnier, Job F. M. van Boven, Thomas Szucs, Daniel Albrecht, Ira Wilson, Sabina De Geest, Marie P. Schneider

**Affiliations:** ^1^ School of Pharmaceutical Sciences, University of Geneva, Geneva, Switzerland; ^2^ Institute of Pharmaceutical Sciences of Western Switzerland, University of Geneva, University of Lausanne, Geneva, Switzerland; ^3^ Nursing Science, Department Public Health, Faculty of Medicine, University of Basel, Basel, Switzerland; ^4^ Department of Hematology, University Hospital of Basel, Basel, Switzerland; ^5^ Research and Development Unit, Institut de Recerca Sant Joan de Déu, Barcelona, Spain; ^6^ Pharmaceutical Care, Department of Pharmaceutical Sciences, University of Basel, Basel, Switzerland; ^7^ Swiss Centre for International Health, Swiss Tropical and Public Health Institute, University of Basel, Basel, Switzerland; ^8^ Patient-as-Partner Project, Geneva University Hospitals, Geneva, Switzerland; ^9^ Innosuisse, Bern, Switzerland; ^10^ Real World Data Strategy and Innovation, Novartis Pharma AG, Basel, Switzerland; ^11^ Faculty of Biology and Medicine, University of Lausanne, Lausanne, Switzerland; ^12^ Department of Clinical Pharmacy and Pharmacology, University Medical Center Groningen, University of Groningen, Groningen, Netherlands; ^13^ Medication Adherence Expertise Center of the Northern Netherlands (MAECON), Groningen, Netherlands; ^14^ European Center of Pharmaceutical Medicine, University of Basel, Basel, Switzerland; ^15^ Federal Department of Home Affairs, Federal Office of Public Health Division, Bern, Switzerland; ^16^ Department of Health Services, Policy and Practice, Brown University School of Public Health, Providence, RI, United States; ^17^ Academic Center for Nursing and Midwifery, Department of Public Health and Primary Care, KU Leuven, Leuven, Belgium

**Keywords:** medication adherence, multi-level interventions, priority setting, COST action ENABLE, Swiss research, implementation science, interprofessionality, healthcare policy

## Introduction

Medication non-adherence, i.e., patients not taking medications as prescribed, diminishes clinical outcomes and quality of life. This phenomenon is endemic, leading to an estimated 200,000 premature deaths and 125 billion Euros of excess healthcare costs each year in Europe alone [1].

Medication adherence (MA) consists of three interrelated phases: initiation (first dose taken), implementation (daily regimen management) and discontinuation (last dose taken) [2]. In Switzerland, half of the population aged 15 years and older takes at least one medication per week [3]. Worldwide, studies showed that most patients (69–96%) initiate their prescribed treatments correctly, but 30–50% do not implement them optimally. Within 2 years of their first prescription, more than 50% of patients discontinue use of their prescribed medication [1].

The high prevalence and consequences of medication non-adherence have attracted considerable attention over the past 30 years. Researchers, clinicians and policymakers search for improved methods to assess this phenomenon and its determinants, along with effective MA-enhancing multifaceted interventions tailored to patients’ needs [4].

In 2011, the UK’s National Health Service (NHS) introduced the largest-ever national evidence-based MA intervention: the New Medicine Service (NMS) [5]. After certain new medications are prescribed, NHS pharmacists identify and assess any adherence problems and their causes. While increasing quality-adjusted life-years per patient, the NMS has led to reduced costs compared with standard care [6].

While evidence on addressing medication non-adherence is available, translation of that evidence into real-world clinical practice remains challenging. In Switzerland, no adherence-focused national programs or guidelines have been developed, implemented or evaluated [7].

Therefore, the launch of the European Network to Advance Best practices and technoLogy on medication adherencE (ENABLE), a Cooperation in Science and Technology (COST) Action (https://www.cost.eu/actions/CA19132/) in 2020 was well-timed [8]. Linking stakeholders across 39 European countries, including Switzerland, ENABLE aims to drive the implementation of MA-enhancing technologies into clinical practice.

## Launch of the Swiss COST ENABLE Network

On March 18th, 2022 Switzerland’s ENABLE country group launched a pioneering initiative to set priorities for implementing MA interventions in real-world settings. An online conference was held to: 1) connect Swiss and international stakeholders; and 2) initiate MA priority-setting, particularly regarding implementation of MA interventions and technologies.

Seventy-five participants including researchers, clinicians, healthcare industry delegates and one policymaker, from 34 countries took part. Keynote lectures and an interprofessional roundtable highlighted the state-of-the-art MA understanding regarding the place for digital technology in real-world settings, alongside strategies to address non-adherence. Priorities to move toward a more favourable, multilevel ecosystem for developing, implementing and maintaining MA interventions in Switzerland were discussed. While the highest priority was identified as support to the implementation of MA interventions in real-world settings, education, research and policy measures were also highlighted (Supplementary File S1).

**FIGURE 1:**
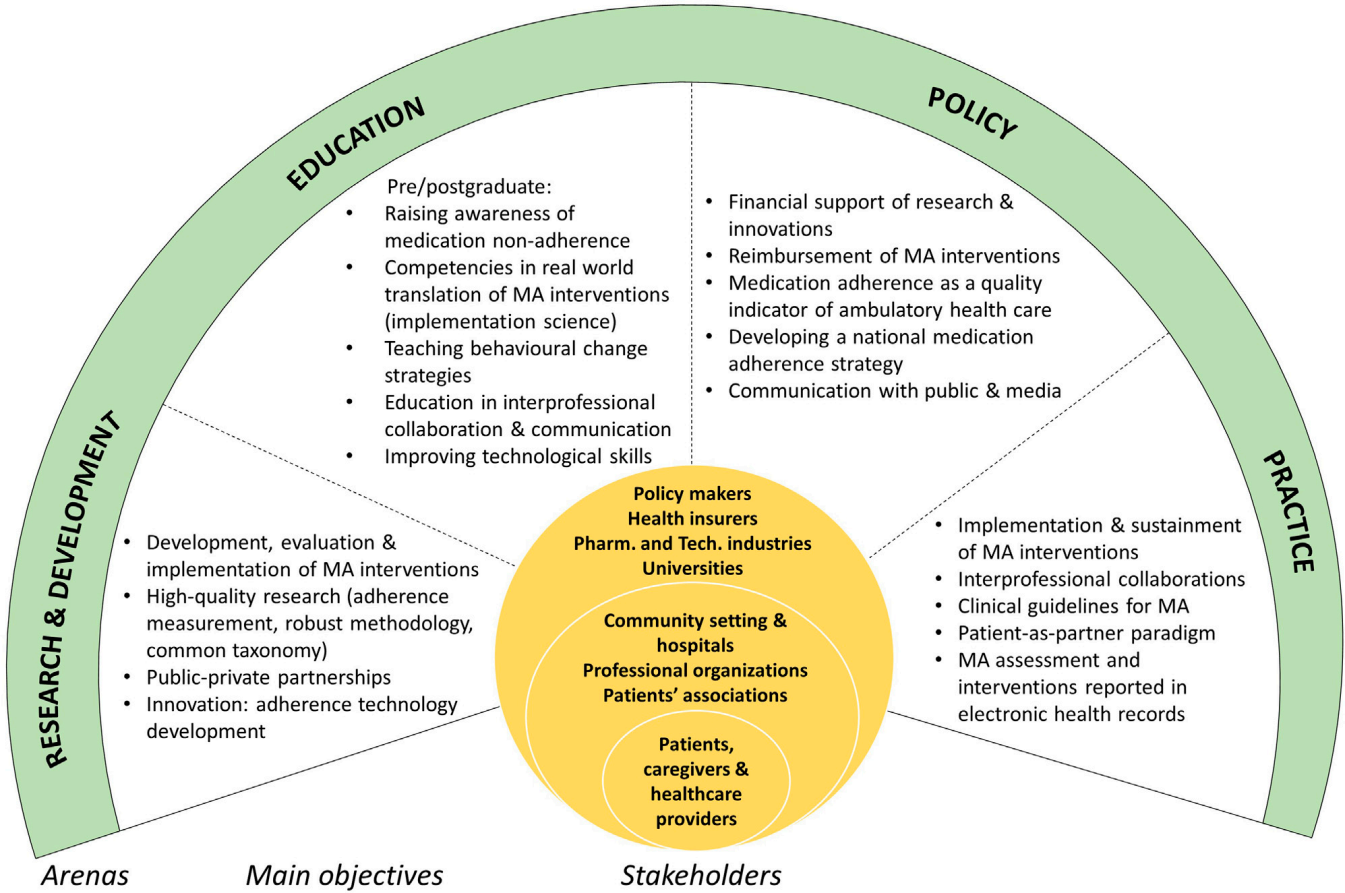
Framework for addressing medication adherence in a complex, multilevel ecosystem: arenas, main objectives and stakeholders. European Network to Advance Best practices and technoLogy on medication adherencE (ENABLE), a Cooperation in Science and Technology (COST) Action, Switzerland, 2022.

The ideas discussed were organized into a framework (Figure 1)—partly for a systematic elaboration of strategies to address MA at a national level, partly to move towards an integrated, patient-as-partner healthcare system.

## Priority Setting to Implement MA Interventions in Switzerland

While MA is a major public health concern that warrants high-priority treatment by health authorities, it remains absent from Swiss policymakers’ agendas. As a first step, raising awareness for MA and promoting research are needed to change the mindset and attitudes of the population, health care professionals (HCPs), and all other stakeholders.

### Prerequisites for MA Intervention Implementation

#### Multilevel Ecosystem

To achieve synergies, stakeholders must work interprofessionally, fine-tuning their actions, and engaging their responsibilities to collaboratively monitor and maximize MA in the ecosystem. Connecting innovative ecosystems in Switzerland (e.g., funding agencies, start-up incubators) and public-private-partnerships will foster further MA development (e.g., new MA technologies).

While reimbursement of MA interventions -by health insurers- will enhance adoption of these interventions in clinical practice, the scientific basis to support reimbursement should go beyond demonstration of MA improvement. A clear value statement is needed in which the effects of MA interventions guarantee favorable clinical outcomes [9].

#### Patient-As-Partner

Along with shared decision-making and therapeutic education, the patient-as-partner paradigm is critical to raise MA awareness and for mutual learning.

However, efforts should not only target the individual level. Building trusting partnerships and communication across all HCPs and patients reinforces MA by decreasing conflicting information and ensuring continuity of care. Emphasizing MA interventions, behavioural training and interprofessional competencies should be integrated within the training curricula of all involved HCPs (i.e., including physicians, nurses, psychologists, pharmacists) to develop competencies vital to MA management in clinical practice. Integrated care models powered by digitalization provide fertile ground for these efforts and innovations.

#### Monitoring Medication Adherence

Measuring MA and its determinants in routine practice enables researchers to know when patients have MA difficulties or underlying non-adherence risk factors and provides an informed basis for clinical decision-making. In routine practice, adherence should be treated both as a patient’s fifth vital sign and as a system-level indicator of quality of care. Technology (e.g., smartphone apps, electronic adherence monitors) can be helpful, but must be designed to match patients’ and HCPs’ preferences.

#### Investing in Translation From Research to Real-World Settings

Internationally, research evidence underscores the value of multilevel interventions, monitoring and equipping HCPs to address MA [1, 4]. However, in Switzerland, implementation of MA interventions in clinical practice is limited. Assisting the uptake of research evidence into clinical practice will require a systematic implementation science approach [10].

### Next Steps to Support the Translation of MA Interventions into Swiss Clinical Practice

First, key interprofessional cross-sectoral stakeholders need to be identified to address MA in the multilevel ecosystem and to determine contextually appropriate multilevel MA interventions. Every step will need to fit within a national Swiss MA strategy.

Second, efforts towards coordinated care models must be strengthened and tailored to facilitate MA management. Technology-based innovations and digital health infrastructure (e.g., electronic medical records, teleconsultation) and use of new technologies (e.g., digital pill boxes, smart inhalers) and methodologies (e.g., artificial intelligence) have the potential to facilitate or contribute directly to interventions. Importantly, technology literacy needs to be concurrently enhanced at the patient and HCP levels.

Third, contextually appropriate implementation strategies (e.g., development of clinical guidelines, patient/consumer involvement) need to be integrated using a multilevel approach.

Fourth, research funding schemes must be adapted to provide opportunities for MA research (e.g., MA is prioritized by the Innovative Medicines Initiative, an EU public-private-partnership). Researchers should be encouraged to expand their foci from traditional efficacy/effectiveness research to include evaluation of implementation strategies for MA interventions in real-world settings.

Finally, to maximize impact, the synergies between bottom-up and top-down approaches, public/private initiatives and interprofessional collaborations are required to power much-needed change.

### Conclusion: A Framework for Change

The Swiss COST ENABLE conference built on its participants’ critical knowledge, competencies and experience of interprofessional dynamics to define priorities to tackle MA as a major public health issue. One point was clear: translating MA-relevant evidence into real-world outcomes demands multilevel approaches.

Through the Swiss ENABLE conference, diverse presenters and participants committed to raising MA awareness. Regarding the knowledge and practice gap, an initial consensus was reached on three early steps: provide the foundations for MA-supportive ecosystems, translate effective interventions into clinical practice and promote research into MA-enhancing technologies. The Swiss strategies discussed here will potentially inform research activities, policies and implementation of MA interventions across Europe.
