# Supplementary material for: Swiss Priority Setting on Implementing Medication Adherence Interventions as Part of the European ENABLE COST Action
Source: Int J Public Health. 2022 Aug 12;67:1605204. doi: 10.3389/ijph.2022.1605204 (PMC9411421; doi:10.3389/ijph.2022.1605204)
Supplement: Supplementary file 1 [file DataSheet1.docx]

**SUPPLEMENTARY FILE S1:** Audience engagement voting results on priority setting (n=34, mean and standard deviation), as part of the conference European Network to Advance Best practices and technoLogy on medication adherencE (ENABLE), a Cooperation in Science and Technology (COST) Action, Switzerland, 2022


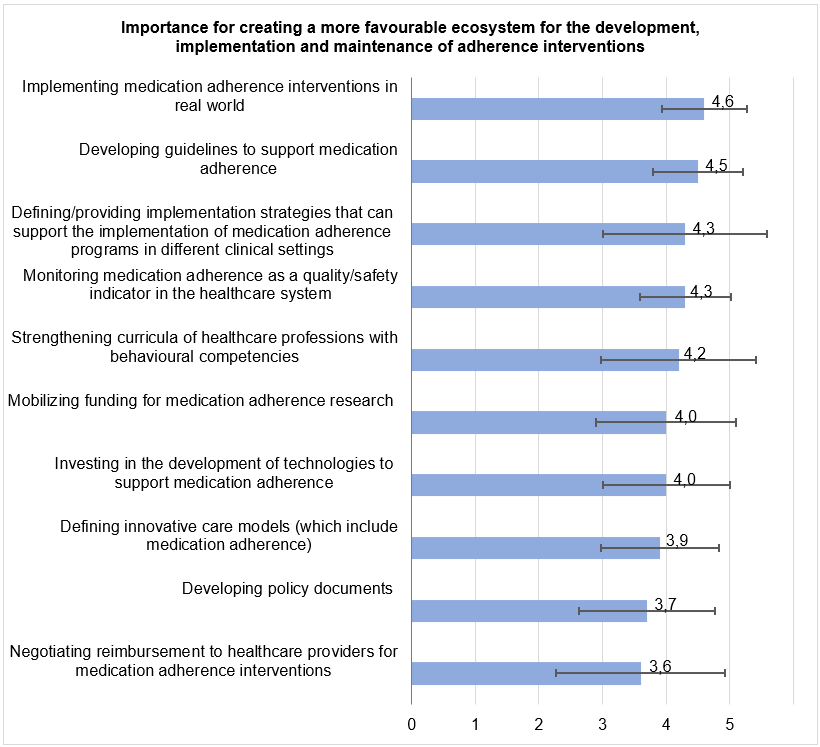


*Note:* Items were answered using a 0 to 5-point Likert scale where 0 = Not important at all to 5 = very important
